# Perceived Sodium Reduction Barriers Among Patients with Chronic Kidney Disease: Which Barriers Are Important and Which Patients Experience Barriers?

**DOI:** 10.1007/s12529-017-9668-x

**Published:** 2017-09-08

**Authors:** Yvette Meuleman, Tiny Hoekstra, Friedo W. Dekker, Paul J. M. van der Boog, Sandra van Dijk, The ESMO study group, Sandra van Dijk, Yvette Meuleman, Friedo W. Dekker, Tiny Hoekstra, Gerjan Navis, Liffert Vogt, Paul J. M. van der Boog, Willem Jan W. Bos, Gert A. van Montfrans, Elisabeth W. Boeschoten, Marion Verduijn, Lucia ten Brinke, Lara Heuveling, Marjolein Storm, Karen Prantl, Anke Spijker, Arjan J. Kwakernaak, Jelmer K. Humalda, Tonnie van Hirtum, Robin Bokelaar, Marie-Louise Loos, Anke Bakker-Edink, Charlotte Poot, Yvette Ciere, Sophie Zwaard, Glenn Veldscholte

**Affiliations:** 1 Department of Health, Medical, and Neuropsychology, Institute of Psychology, Leiden University, Wassenaarseweg 52, 2300 RB Leiden, The Netherlands; 2 Department of Medical Psychology, Leiden University Medical Center, Leiden, The Netherlands; 3 Department of Clinical Epidemiology, Leiden University Medical Center, Leiden, The Netherlands; 4 Department of Nephrology, VU University Medical Center, Amsterdam, The Netherlands; 5 Department of Nephrology, Leiden University Medical Center, Leiden, The Netherlands

**Keywords:** Chronic kidney disease (CKD), Lifestyle adherence, Patient-centered care, Perceived barriers, Reducing sodium intake, Self-management interventions

## Abstract

**Purpose:**

The purposes of this study were to assess the importance of perceived sodium reduction barriers among patients with chronic kidney disease (CKD) and identify associated sociodemographic, clinical, and psychosocial factors.

**Method:**

A total of 156 patients with CKD completed a questionnaire assessing sodium reduction barriers (18 self-formulated items), depressive symptoms (Beck Depression Inventory), perceived autonomy support (Modified Health Care Climate Questionnaire), and self-efficacy (Partners in Health Questionnaire). Factor analysis was used to identify barrier domains. Correlation coefficients were computed to examine relationships between barrier domains and patient characteristics.

**Results:**

Nine barrier domains were identified. Barriers perceived as important were as follows: high sodium content in products, lack of sodium feedback, lack of goal setting and discussing strategies for sodium reduction, and not experiencing CKD-related symptoms (mean scores > 3.0 on 5-point scales, ranging from 1 ‘no barrier’ to 5 ‘very important barrier’). Other barriers (knowledge, attitude, coping skills when eating out, and professional support) were rated as moderately important (rated around midpoint), and the barrier ‘intrinsic motivation’ was rated as somewhat important (mean score = 1.9). Sodium reduction barrier domains were not associated with gender and kidney function, but were associated with age, level of education, number of comorbidities, perceived autonomy support, depressive symptoms, and self-efficacy (range *r* = 0.17–0.35). Patients with lower self-efficacy and perceived autonomy support scores experienced most sodium reduction barriers.

**Conclusion:**

Patients with CKD experience multiple important sodium reduction barriers and could benefit from support strategies that target various sodium reduction barriers and strengthen beliefs regarding self-efficacy and autonomy support. Additionally, environmental interventions should be implemented to reduce sodium levels in processed foods.

## Introduction

In patients with chronic kidney disease (CKD), kidney function gradually and usually permanently declines over time. The severity of CKD can be classified into five stages, with CKD stage 5 as most advanced stage of CKD. In CKD stage 5, many patients progress towards end stage kidney disease (ESKD) in which kidney replacement therapy (i.e., transplantation or dialysis) becomes necessary to prolong life [1]. In all stages of CKD, patients are being advised to reduce their sodium intake to a maximum of 2000 mg a day [2] because a reduction to the recommended amount of sodium has been associated with beneficial health outcomes, for example a decreased blood pressure [3–5]. In early stages of CKD, limiting sodium intake is considered especially important because it can contribute to slowing down disease progression towards ESKD and to reduce risks of cardiovascular complications [6, 7]. Unfortunately, daily sodium consumption of most patients with CKD far exceeds the advised sodium intake [5], and it seems that current health care does not provide patients with the necessary support to incorporate the sodium treatment guidelines into their daily life.

Literature suggests that in order to successfully limit dietary sodium in patients with CKD, behavioral support strategies are needed [3, 5]. It has also been argued that individualization of support is vital, for instance by taking patients’ perceived barriers into account [8, 9]. However, to the best of our knowledge, there is a paucity of data on barriers regarding a low-sodium diet in patients with CKD, and the studies that have been conducted were performed in patients with ESKD (more specifically, in patients receiving hemodialysis) [10–12]. Moreover, these studies focused on only a few sodium barriers; Clark-Cutaia et al. assessed the importance of three sodium barrier items (‘Sometimes I crave salty foods’, ‘Resisting salty foods where I work is difficult for me’, and ‘I have trouble keeping track of the amount of the nutrients that I eat from meal to meal (such as sodium, potassium, phosphorus)’ [13]) [10], and Agondi et al. and Welch et al. assessed the importance of five sodium barrier items (i.e., ‘Eating a low salt diet makes it difficult to eat out’, ‘The food does not taste good in a low salt diet’, ‘It is expensive to follow a low salt diet’, ‘Following a low salt diet takes too long’, and ‘It is very difficult to understand how to follow a low salt diet’) [11, 12].

To increase our understanding of the sodium reduction barriers patients encounter in earlier stages of CKD (i.e., CKD stage 1 to 4), our study group conducted a qualitative study (i.e., focus groups with patients and health care professionals) and the results indicate that patients with CKD experience multiple barriers when reducing sodium, including a lack of intrinsic motivation, knowledge, personal goal setting and action planning, feedback, coping skills, and support [14]. This study provided in-depth knowledge about patients’ experiences when reducing sodium, but further research is warranted to assess how common these identified barriers are in patients with CKD and to examine which barriers patients perceive as most important. Furthermore, the qualitative design is not suitable for assessing which factors are associated with sodium reduction barriers in order to identify patients that will benefit most from support strategies. Previous studies have shown that patients with ESKD who received hemodialysis treatment for a longer time experience more sodium reduction barriers [11] and that sodium intake is associated with age, gender, level of education, and comorbidities [10, 11, 15, 16]. Additionally, literature suggests that psychosocial factors, such as depressive symptoms [17], self-efficacy [18, 19], and support from health care professionals [18] are associated with treatment adherence in patients with ESKD, and hence, it is plausible that these factors are also related to difficulties with adhering to the sodium treatment guidelines.

Therefore, the objectives of this study were to assess the importance of previously identified sodium reduction barriers among patients with CKD stage 1 to 4 and to investigate whether sociodemographic, clinical, and psychosocial factors were associated with perceived sodium reduction barriers. This knowledge will enable us to develop individualized behavioral strategies to support patients with CKD in reducing sodium intake and consequently slow down disease progression towards ESKD.

## Methods

### Design and Participants

Participants of this cross-sectional study were recruited between November 2013 and February 2014 from Leiden University Medical Centre in the Netherlands. Dutch speaking patients who were treated for their kidney disease by a nephrologist and with a kidney function (estimated Glomerular Filtration Rate [eGFR]) of at least 20 ml/min/1.73 m^2^ (i.e., no upper limit for eGFR) were eligible for inclusion. Patients in CKD stage 5 in need for or receiving kidney replacement therapy or conservative therapy (i.e., palliative care) were excluded to limit heterogeneity in treatment characteristics that may influence dietary behavior. Eligible patients received a study invitation, detailed information explaining the procedure and confidentiality, an informed consent form (for study participation and medical data collection), a questionnaire, and a pre-stamped envelope. Participating patients returned the signed informed consent form and the completed questionnaire. Approval of the medical ethics committee was obtained (P10.056).

### Measurements

After receiving signed informed consent forms, clinical data was collected from hospital information systems and medical records. The most recent medical measurements were included, given that measurements were conducted within the prior year. Kidney function was calculated using the abbreviated Modification of Diet in Renal Disease formula [20]. Sodium excretion (i.e., a measure for dietary sodium intake) and protein excretion were estimated from 24-h urinary samples. The number of comorbidities was computed based on the presents of diabetes mellitus (type 1 or 2) and cardiovascular disease (cerebrovascular accident, coronary artery disease, or peripheral artery disease).

The questionnaire comprised items addressing patients’ experiences with sodium reduction and sociodemographic and psychosocial factors. Prior to usage, the questionnaire was pilot tested among nine patients and revised based on feedback regarding acceptability and feasibility. *Sodium reduction* patients were asked whether they had received a sodium advice from professionals and whether they (had) tried to reduce their sodium intake. If patients indicated having experience with reducing sodium (i.e., irrespective of whether they succeeded), they were invited to fill out items regarding experiences with reducing sodium. The questionnaire also assessed *perceived sodium adherence* (single item using Visual Analogue Scale [VAS], ranging from 1 ‘never’ to 10 ‘always’) and *perceived barriers for reducing sodium intake* (using 18 self-formulated items based on a previous qualitative study [14], rated on a five-point scale ranging from 1 ‘no barrier’ to 5 ‘very important barrier’). A sum score of all sodium reduction barrier items was computed as indication for the amount of difficulties experienced. *Depressive symptoms* were measured using the Beck Depression Inventory (BDI) [21]. The BDI contains 21 items using a four-point scale (0–3), and total scores ranged from 0 to 63, with higher scores indicating more depressive symptoms. The BDI proved to be reliable with a Cronbach alpha value of 0.88. *Self-efficacy regarding self-management skills* was assessed using the Partner in Health questionnaire (PIH) [22] and measured the following domains: knowledge of disease, active participating in decision-making, ability to monitor and manage symptoms, adopt a healthy lifestyle, and manage physical, emotional, and social consequences. A total score was calculated based on the sum of 13 items rated on a nine-point scale (ranging from 0 ‘very bad’ to 8 ‘very good’). The total score ranged from 0 to 104, with a higher score indicating a higher self-efficacy. The PIH showed good reliability with a Cronbach alpha value of 0.89. *Perceived autonomy support from health care professional* was assessed using the Modified Health Care Climate Questionnaire (HCCQ) [23]. Six items were rated on a seven-point scale (ranging from 1 ‘fully disagree’ to 7 ‘fully agree’). A total score was computed by averaging item scores, and a higher score implied greater perceived support from professionals regarding being autonomous. The HCCQ showed good reliability with a Cronbach alpha value of 0.88.

### Analysis

Descriptive statistics were computed for patient characteristics and sodium reduction barriers. Chi-square tests of association and *t* tests were conducted to detect differences in patient characteristics between patients who were included in and excluded from analyses.

For the purpose of data reduction, exploratory factor analysis (EFA) using varimax rotation was conducted to identify underlying sodium reduction barrier domains. The number of factors extracted was based on examination of a scree plot, Kaiser criterion (eigenvalues > 1), and the (theoretical) interpretability of the extracted factors. Furthermore, data was inspected for several standard indices to assess the factorability and the strength of the relationship among items, including sample adequacy (e.g., Kaiser-Meyer-Olkin measure > 0.50), sphericity (i.e., a significant Bartlett’s test [*p* < 0.05]), common variance (i.e., communalities > 0.5), correlations (e.g., no multicollinearity), and factor loadings (e.g., minimum item loading of 0.30, and no or few cross-loadings [i.e., item loading of 0.32 or higher on two or more factors]) [24, 25]. Subscales were created by averaging items, and internal consistency was examined using Cronbach’s alpha measures. Correlation coefficients were calculated to test associations between sodium reduction barriers (separate barrier domains and barrier sum score) and sociodemographic (age, gender, and level of education), clinical (kidney function and number of comorbidities), and psychosocial factors (perceived autonomy support, depressive symptoms, and self-efficacy): Pearson correlation coefficients for continuous factors and point-biserial correlation coefficient for dichotomous factors. To avoid biased results and loss of power, missing data were imputed using multiple imputation (using 10 repetitions)—a recommended technique to deal with missing data in which plausible estimates are calculated based on known patient characteristics [26, 27].

Several sensitivity analyses were conducted to test the robustness of our results. First, analyses were repeated without imputing missing data. Second, Spearman’s rank-order correlation coefficients were calculated to investigate if results would change when treating ‘level of education’ and ‘number of comorbidities’ (i.e., no comorbidity, 1 comorbidity, and 2 comorbidities) as ordinal variables (i.e., 6 education categories ranging from ‘elementary education’ to ‘higher professional education/university’ instead of 2 categories [low and high]). Third, *t* tests and one-way analyses of variance (ANOVA Tukey post hoc) were used to determine if barriers differed between categories of gender, level of education, and number of comorbidities. Fourth, *p* values of correlation coefficients were corrected for multiple testing using Benjamini and Hochberg False Discovery Rate [28]. Finally, to investigate if the amount of difficulties patients encounter when reducing sodium intake is also an indication of adherence to the sodium treatment guidelines, Pearson correlation coefficients were calculated for the associations between the barrier sum score and sodium adherence using objective (i.e., 24-h urinary sodium excretion) and subjective (i.e., perceived sodium adherence) measures. All analyses were performed using SPSS 24.0, and *p* values of < 0.05 were considered statistically significant.

## Results

### Patient Characteristics

**Fig. 1:**
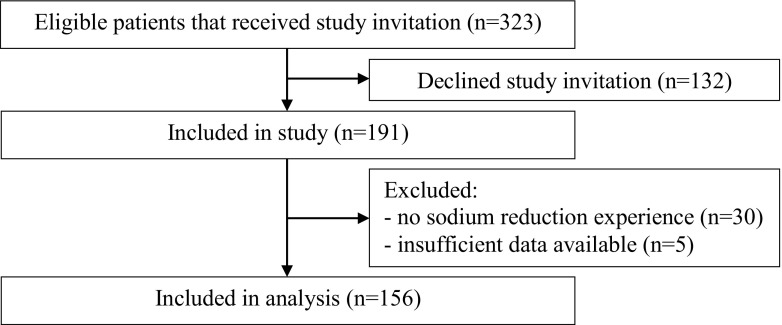
Flow diagram of the study

The questionnaire was returned by 191 out of 323 patients (59.1%), after which 35 patients (18.3%) were excluded from analysis because they had no experience with reducing sodium intake or because only sociodemographic data was available (see Fig. 1). No significant differences in patient characteristics (see Table 1) were detected between patients who were included in and excluded from the analyses, with the exception that included patients had lower levels of hemoglobin (*t* = −2.3, *p* = .024), lower kidney function (*t* = −2.5, *p* = .019), and more often cardiovascular disease (χ^2^ = 6.3, *p* = .012). In the included sample of 156 patients, the mean (SD) sodium excretion was 145.7 (60.1) mmol/24-h, and patients gave themselves a mean (SD) mark of 6.6 (2.7) out of 10 on perceived sodium adherence. Table 1 depicts all patient characteristics.

**Table 1:** Patient characteristics (*n* = 156)

Characteristic	
Sociodemographic	
Age, years, mean ± SD	62.1 ± 13.6
Sex, male, *N* (%)	93 (59.6)
Ethnicity, Dutch, *N* (%)^*^	139 (89.1)
Married or cohabiting, yes, *N* (%)	118 (75.6)
Education, low, *N* (%)^**,a^	86 (55.1)
Paid work status, yes, *N* (%)^***^	53 (34.0)
Clinical ^b^	
Primary cause of renal failure, *N* (%)^□,c^	
Diabetes mellitus	5 (3.2)
Glomerulonephritis	44 (28.2)
Renal vascular disease	16 (10.3)
Other cause	79 (50.6)
Diabetes mellitus, *N* (%)^□^	27 (17.3)
Cardiovascular disease, *N* (%)^□^	50 (32.1)
Time on nephrology care, years, median (IQR)^□□^	4 (1–13)
Systolic blood pressure, mm Hg, mean ± SD^□□□^	134 ± 19
Diastolic blood pressure, mm Hg, mean ± SD^□□□^	79 ± 11
Use of antihypertensive medication, yes, *N* (%)^∆^	123 (78.8)
Body mass index, kg/m^2^, mean ± SD^∆∆^	27.1 ± 4.4
Hemoglobin, g/dL, mean ± SD^∆∆∆^	13.5 ± 1.6
eGFR, mL/min/1.73 m^2^, mean ± SD^†^	47.6 ± 21.5
Protein excretion, g/24-h, median (IQR)^‡^	0.3 (0.1–0.9)
Sodium^b^	
Sodium, mmol/24-h, mean ± SD^‡,d^	145.7 ± 60.1
Perceived sodium adherence, mean ± SD^ϕ^	6.6 ± 2.7
Received sodium advice from professional, yes, *N* (%)	67 (42.9)
Psychosocial	
Perceived autonomy support, mean ± SD^ϕϕ^	5.8 ± 0.8
Self-efficacy, mean ± SD^ϕϕϕ^	73.7 ± 17.5
Depressive symptoms, mean ± SD^⋄^	7.3 ± 6.4

Continuous variables are presented as mean ± SD for normally distributed variables andas median (IQR) for skewed variables

Available for: ^*^155 (99.4%), ^**^154 (98.7%), ^***^153 (98.1%), ^□^144 (92.3%), ^□□^140 (89.7%), ^□□□^143 (91.7%), ^∆^127 (81.4%), ^∆∆^111 (71.2%), ^∆∆∆^115 (73.7%), ^†^114 (73.1%), ^‡^78 (50.0%), ^ϕ^144 (92.3%), ^ϕϕ^141 (90.4%), ^ϕϕϕ^86 (55.1%), ^⋄^138 (88.5%) patients

^a^Low education was classified as primary education and lower secondary education

^b^Differences in time between completing the questionnaire and clinical measurements/mean (SD) of 0.5 (2.5) months for eGFR, mean (SD) of 1.7 (3.3) months for sodium excretion, mean (SD) of 1.6 (3.7) months for protein excretion, mean (SD) of 0.7 (0.9) for hemoglobin, median (IQR) of 3.6 (0.4–9.9) months for body mass index, and median (IQR) of 0.2 (−1.3–3.3) months for blood pressure measurements

^c^Primary kidney disease was classified into four categories following the European Renal Association-Dialysis and Transplantation Association registry codes [29]

^d^Conversion factor for mmol/24-h sodium excretion to mg/24-h sodium excretion: ×23

### Perceived Sodium Reduction Barrier Scores

Only four patients (2.6%) indicated that they experience no barriers when reducing sodium. The results showed that various sodium reduction barriers could be considered important (mean scores > 3.0 on a 5-point scale). Patients had problems managing their low-sodium diet because many products contain (high levels of) sodium, they did not experienced CKD-related symptoms, they lack feedback on their sodium intake, and they did not set personal sodium goals and discuss strategies on how to reduce sodium with professionals. Sodium reduction barriers that were considered somewhat important were motivation-related barriers (mean scores ≤ 2.0), indicating that patients did not experience difficulties because they believe reducing sodium is not beneficial for them personally or their health. The remaining nine barrier items were rated as moderately important (mean scores ranging from 2.2 to 2.8). Table 2 contains scores of all barrier items.

**Table 2:** Descriptives of sodium reduction barrier items, domains, sum score, and Chronbach alpha values (*n* = 156)

Sodium reduction barriers	Mean (SD)
Attitude (α = 0.63)^□^	2.5 (0.8)
Low-sodium food taste bad	2.7 (1.2)
A low-sodium diet is unsocial	2.4 (1.1)
A low-sodium diet is time and energy consuming	2.3 (1.1)
Low-sodium products are expensive	2.6 (1.2)
Symptoms (α = 0.86)^□□^	3.2 (1.2)
Not feeling ill	3.3 (1.2)
No CKD-related symptoms	3.0 (1.2)
Professional support (α = 0.87)^□□□^	2.5 (0.9)
Health care professionals are not patient-centered enough	2.4 (0.9)
Health care professionals have insufficient time to support me	2.6 (0.9)
Knowledge (α = 0.75)^∆^	2.3 (0.9)
Insufficient knowledge on how to reduce sodium intake	2.2 (1.0)
Insufficient knowledge about sodium content in products	2.4 (1.0)
Intrinsic motivation (α = 0.83)^□□^	1.9 (0.8)
A low-sodium diet is not beneficial for my health	1.9 (0.9)
A low-sodium diet is not important for me personally	2.0 (0.8)
Feedback (α = 0.70)^∆∆^	3.1 (1.1)
Insufficient insight into my daily sodium intake	3.3 (1.2)
Receiving insufficient feedback on my sodium intake	3.0 (1.2)
Goal and strategy (α = 0.62)^∆∆^	3.4 (1.0)
No personal and concrete goals have been set to reduce my sodium intake	3.1 (1.2)
No sodium reduction strategies have been discussed with my professional	3.7 (1.1)
Coping skills when eating out^∆∆∆^	
Difficult to refuse food at parties and when eating out	2.8 (1.2)
Sodium in products^□□□^	
The majority of products contain (high levels of) sodium	3.5 (1.2)
Barriers sum score (α = 0.79)^a^	49.5 (9.2)

Data available between 135 (86.5%) and 147 (94.2%) patients for each single barrier

Loadings were strong for primary items in the domains ‘attitude’ (0.88, 0.78, 0.62, and 0.86, respectively), ‘symptoms’ (0.91 and 0.91), ‘professional support’ (0.90 and 0.92), ‘knowledge’ (0.83 and 0.90), ‘intrinsic motivation’ (0.91 and 0.88), ‘feedback’ (0.76 and 0.84), ‘goal and strategy’ (0.84 and 0.87), ‘coping skills when eating out’ (0.53), and ‘sodium in products’ (0.82)*—*all other item loadings on the factors were well below 0.40

Data of domains available for the following: ^□^143 (91.7%), ^□□^139 (89.1%), ^□□□^129 (82.7%), ^∆^137 (87.8%), ^∆∆^141 (90.4%), ^∆∆∆^135 (86.5%). Possible range is 1–5, with 1 indicating ‘no barrier’ and 5 ‘very important barrier’

^a^Barrier sum score was calculated when all barrier data were available (*n* = 111, 71.2%). Possible score range is 18–90, with higher scores indicating more difficulties with reducing sodium intake

### Perceived Sodium Reduction Barrier Domains

The EFA showed a nine factor solution, explaining 81.8% of the variance. Overall, indicators showed that our data was suitable for factor analysis: no multicollinearity was detected, Kaiser-Meyer-Olkin exceeded the minimum of 0.50 (value = 0.63), Bartlett’s test of sphericity was significant (*p* = .000), and communalities were greater than 0.50 (mean value = 0.82). Seven domains with adequate eigenvalues (> 1) were created that consisted of multiple items with high item loadings (i.e., ranging from 0.62 to 0.92): attitude (4 items), professional support (2 items), symptoms (2 items), knowledge (2 items), intrinsic motivation (2 items), feedback (2 items), and goal and strategy (2 items). The domains also showed moderate to good reliability with Chronbach alpha values ranging from 0.62 to 0.87. The interpretation of the two remaining factors was less clear with freestanding items assessing the barriers ‘sodium in products’ and ‘coping skills when eating out’, and with borderline cross-loading detected in the latter barrier (i.e., with the domain ‘attitude’). Solutions were examined, but did not change the results. Due to the nature of this EFA (i.e., data reduction in light of correlation analysis) and the importance of both items (mean scores were 2.8 for ‘coping skills when eating out’ and 3.5 for ‘sodium in products’), the decision was made to treat these single items as separate barrier domains. The barrier ‘sodium in products’, however, was excluded from the correlation analysis because sodium content of products cannot be modified by patients, and hence, is most likely not associated with patient characteristics. Table 2 depicts all barrier domains and Chronbach alpha values.

### Factors Associated with Perceived Sodium Reduction Barrier Domains

The correlation coefficients showed that several sociodemographic, clinical, and psychosocial factors were associated with sodium reduction barrier domains (see Table 3). First, associations were found between self-efficacy and the barriers ‘knowledge’, ‘attitude’, and ‘feedback’: patients who believed to a lesser extent that they are capable of managing their disease reported more often that a lack of knowledge was a barrier to reduce sodium intake (*p* = .006), expressed a more negative attitude towards limiting sodium (*p* = .036), and more often considered a lack of feedback as a barrier (*p* = .049). Associations were also found between age, the number of comorbidities, depressive symptoms, and the barrier ‘symptoms’: older patients, patients with more comorbidities, and patients with less depressive symptoms more often reported that not experiencing CKD-related symptoms was a barrier for reducing sodium (*p* = .015, *p* = .027, and *p* = .007, respectively). Furthermore, age and level of education were associated with the barrier ‘goal and strategy’: younger patients and patients with high levels of education more often considered a lack of setting goals and planning strategies as a barrier for reducing sodium (*p* = .012 and *p* = .003). The barrier ‘coping skills when eating out’ was associated with number of comorbidities and self-efficacy: a lack of skills when eating out as barrier for reducing sodium was more often mentioned by patients with more comorbidities (*p* = .035) and by patients who believe to a lesser extent they are capable to manage their disease (*p* = .044). Furthermore, associations were also found between the barrier ‘professional support’, perceived autonomy support, and self-efficacy: patients who experienced insufficient professional support as a barrier believed to a lesser extent that they receive sufficient autonomy support from professionals and believed to a lesser extent that they are capable to manage their disease (*p* < .001 and *p* = .005). No factors were associated with the barrier ‘intrinsic motivation’, and gender and kidney function were not related to barrier domains. Finally, perceived autonomy support and self-efficacy were associated with the barrier sum score: patients who believed to a lesser extent that they receive sufficient autonomy support from professionals and patients who believed to a lesser extent that they are capable to manage their disease, experienced more barriers when limiting sodium intake (*p* = .032 and *p* = .013).

**Table 3:** Correlation coefficients for perceived barriers and sociodemographic, clinical, and psychosocial characteristics (*n* = 156)

Sodium reduction barriers	Age^a^	Gender^b^	Level of education ^b^	Number of comorbidities^a^	eGFR^a^	Autonomy support^a^	Depressive symptoms^a^	Self-efficacy^a^
Attitude	−.06	.17	−.07	.10	−.02	−.01	.08	−*.18* ^*^
Symptoms	*.21* ^*^	−.01	.11	*.19* ^*^	−.16	−.02	−*.25* ^**^	−.09
Professional support	−.02	.07	.05	.04	.00	−*.35* ^**^	.09	−*.25* ^**^
Knowledge	−.07	.03	−.04	.08	.04	−.10	.06	−*.25* ^**^
Intrinsic motivation	−.10	−.04	.11	−.18	.12	−.13	−.11	−.06
Feedback	−.16	.10	−.01	.11	.00	−.15	.03	−*.17* ^*^
Goal and strategy	*−.24* ^*^	−.03	*.25* ^**^	−.03	.13	−.12	−.06	.11
Coping skills when eating out	−.05	.08	−.02	*.19* ^*^	.12	−.14	.09	−*.19* ^*^
Barriers sum score	−.10	.03	.09	.11	.03	−*.21* ^*^	−.06	−*.24* ^*^

^a^Pearson correlation coefficients

^b^Point-biserial correlation coefficients for gender and level of education (categories were low and high level of education)

^*^ *P* < 0.05, ^**^ *P* < 0.01

### Sensitivity Analysis

The sensitivity analysis showed that the majority of the results remained stable when conducting complete case analysis, with the exception that no significant relationships were found between self-efficacy and the barriers ‘attitude’ and ‘coping skills when eating out’ (*r* = −.16 [*p* = .164] and *r* = −.19 [*p* = .097]). However, a significant association was now found between perceived autonomy support and the barrier ‘coping skills when eating out’ (*r* = −.18, *p* = .041). Furthermore, compared to the main analysis, similar results were found when calculating Spearman’s rank-order correlation coefficients for associations between sodium reduction barriers, level of education, and number of comorbidities on an ordinal level (data not shown). The *t* tests revealed that patients with high levels of education believed to a higher extent that not setting goals and discussing strategies are important barriers compared to patients with low levels of education (mean [SD] of 3.7 [0.8] compared to 3.2 [1.0], *p* = .003) and revealed that none of the barriers differed between men and women (data not shown). The ANOVAs showed that there was an association between the number of comorbidities and the barrier ‘symptoms’ (*F* = 4.1 [2, 154], *p* = .017), and post hoc comparison indicated that patients with two comorbidities believed to a higher extent that not experiencing CKD-related symptoms is an important barrier compared to patients with no comorbidities (mean [SD] was 3.8 [1.0] compared to 2.7 [1.2], *p* = .013). Furthermore, after correction for multiple testing, two associations remained significant: level of education was associated with the barrier ‘goal and strategy’ (*p = .*036), and perceived autonomy support was associated with the barrier ‘professional support’ (*p* < .001). Only trends were now observed for associations between self-efficacy and the barriers ‘knowledge’ (*p* = .060), and ‘professional support’ (*p* = .055), and between depressive symptoms and the barrier ‘symptoms’ (*p* = .065). Finally, the analyses showed that the barrier sum score was associated with perceived sodium adherence (*r* = −0.53, *p* < .001, *n* = 109), but not with 24-h urinary sodium excretion (*r* = 0.03, *p* = .811, *n* = 60).

## Discussion

This study has shown, in accordance with previous studies [10–12, 14, 30], that patients with CKD perceive adhering to a low-sodium diet as a difficult task. They regard multiple barriers to be important when reducing sodium: patients believed to a high extent that reducing sodium is difficult because many products contain (high levels of) sodium, they do not experience CKD-related symptoms, lack feedback on sodium intake, and do not set sodium goals and discuss strategies for reducing sodium with professionals. Patients also believed to a moderate extent that reducing sodium is difficult because they have insufficient knowledge on how to reduce sodium intake, lack the skills to refuse food when eating out, receive insufficient support from professionals, and perceive a low-sodium diet as untasteful, unsocial, expensive, and time and energy-consuming. Finally, most patients did not experience difficulties because they believe that reducing sodium intake is not beneficial for them personally or for their health. Direct comparison with literature is difficult because previous studies assessed the importance of only a few sodium reduction barriers in patients with ESKD (more specifically, in patients receiving hemodialysis) [10–12]. However, barrier items included by Agondi et al. and Welch et al. partially overlap with items of our domains ‘attitude’, ‘knowledge’, and ‘coping skills when eating out’ [11, 12]. Comparison of mean scores on these items suggests that in all three studies, many patients believed to a moderate extent that reducing sodium is difficult because it is time-consuming and expensive, and they lack knowledge on how to reduce sodium (i.e., items rated around midpoint). However, patients in our study believed to a lesser extent that a bad taste of low-sodium food makes it difficult to adhere to a low-sodium diet (2.7 versus 3.8 and 3.3) [11, 12] and they reported less problems with adherence when eating out (2.8 versus 3.7) [11]. Although all studies specifically address sodium barriers, higher scores in previous studies could be explained by the patient population: patients receiving hemodialysis have additional diet restrictions (e.g., protein and potassium restrictions), and hence, restrict patients even more in choosing and cooking healthy but also tasty food. Finally, previous studies did not assess barriers related to motivation; however, they did assess patient beliefs about benefits of a low-sodium diet [11, 12]. They found that patients with ESKD believed to a high extent that a low-sodium diet is good for their health, which corresponds with our finding that patients do not experience difficulties because they think it is not beneficial for their health.

We also identified patient characteristics that were related to barriers for adhering to a low-sodium diet. Various sodium reduction barriers (i.e., a negative ‘attitude’, and a lack of ‘knowledge’, ‘symptoms’, ‘goal setting and discussing strategies’, ‘feedback’, ‘coping skills when eating out’, and ‘professional support’) were associated with patient characteristics, namely age, level of education, depressive symptoms, number of comorbidities, perceived autonomy support, and self-efficacy. Furthermore, patients who believed to a lesser extent that they are capable to manage their disease and patients who believed to a lesser extent they receive sufficient autonomy support from professionals, experienced more barriers when limiting dietary sodium. These findings suggest that patients differ in the extent to which they experience barriers when striving to adhere to a low-sodium diet. Hereby, this study adds to previous studies that found these sociodemographic and clinical factors associated with sodium excretion [10, 11, 15, 16] and found these psychosocial factors associated with treatment adherence in patients with ESKD [17–19]. Furthermore, the factor that was most consistently related to sodium reduction barriers was self-efficacy, and these findings are in line with a previous study by Curtin et al., who found patients’ self-efficacy, compared to demographic and health-related factors, to be most consistently related to self-management behavior in patients with CKD [31].

### Strengths and Limitations

To the best of our knowledge, this is the first study to assess the importance of perceived sodium reduction barriers in an early phase of CKD and to identify which patients could benefit most from support strategies. Other strengths of this study are that barrier items in the questionnaire were based on a previous qualitative study [14] and that the questionnaire included a broad range of sodium reduction barriers and showed moderate to good psychometric qualities. However, before this questionnaire can be used in practice to identify sodium reduction barriers in individual patients, additional research is needed (e.g., conducting a confirmative factor analysis in a larger population or executing a longitudinal study to evaluate the test-retest reliability).

A limitation of this study is that the date of clinical measurements did not coincide with the date questionnaires were filled out. However, we do not believe this led to biased results as the mean (SD) difference between kidney function measurements and completing the questionnaire was 0.5 (2.5) months and it is expected that kidney function will be constant in this short period of time. There was also missing data, for example because patients did not fully complete the questionnaire, patients did not gave permission to collect medical data, or clinical measurements were not conducted within the prior year. However, the majority of the results prior and after imputations were similar and few differences were detected between patients who were included in and excluded from analyses, and therefore, it is unlikely that missing data led to biased results. Furthermore, the sensitivity analysis showed that the barrier sum score was associated with perceived sodium adherence, but not with 24-h urinary sodium excretion. Explanations for these findings could be that limited data on sodium excretion was available (50%), and that we used a single sodium measurement [9] that was measured on a date that do not coincide with the date questionnaires was filled out (mean [SD] difference was 1.7 [3.3] months). Finally, although our response rate of 59.1% is similar to the average response rate of 65% for postal questionnaires in health care settings [32], it should be noted that our results may not be fully representative of the broad population of patients with CKD.

### Clinical Implications

The results showed that patients with CKD believed to a relatively high extent that limiting dietary sodium is beneficial, but still experience multiple barriers when reducing sodium intake. Therefore, solely educating patients about the benefits of sodium reduction will be insufficient but a multifaceted approach that target various important barriers is required to provide patients with the support they need to incorporate the sodium treatment guidelines into their daily life. The findings also suggest that patients who believed to a lesser extent that they are capable to manage their disease and receive sufficient autonomy support from professionals, experienced more sodium reduction barriers, and thus, support strategies should also strengthen beliefs regarding self-efficacy and autonomy support. The need for such a multifactorial approach to support patients with the complex task of successful behavior change is in line with literature suggesting that theory-based self-regulation interventions that encompass multiple behavior change techniques are required [33–36]. Based on our findings, we believe that in particular, the following strategies could be of use. First, professionals could use the patient-centered techniques of motivational interviewing to elicit behavior change, increase autonomy support, and strengthen patients’ self-efficacy [37, 38]. Additionally, it may provide a solution for the barriers ‘professional support’ and ‘goal and strategy’, as shared decision making, goal setting, and discussing action plans are key elements of motivation interviewing [37]. Second, coaching is considered important in behavior change [39], and continuous support and guidance from professionals could increase self-efficacy and knowledge of patients. Third, an intervention comprising educational cooking sessions successfully reduced sodium intake in patient with CKD [40] and could increase patients’ practical knowledge and skills on how to reduce sodium and positively change attitudes (i.e., cooking flavorful dishes that take little time and are inexpensive). Fourth, self-monitoring has been identified as a key element for successful behavior change [33, 35], and stimulating patients to engage in self-monitoring (e.g., sodium intake by means of an online food diary and home-based blood pressure measurements) could give patients direct feedback on their sodium intake and disease progression. Fifth, sodium content of products and eating out were identified as important barriers, and hence, environmental interventions should be implemented as well (e.g., reducing sodium content in processed and catered foods) [41]. Finally, future studies are needed to investigate whether the suggested strategies can indeed help patients to reduce sodium intake and improve health outcomes in patients with CKD.

### Conclusion

Patients with CKD experience multiple important barriers when reducing sodium intake, especially patients with lower self-efficacy. Addressing perceived sodium reduction barriers could be a starting point for interventions to support patients with CKD in reducing sodium intake and consequently slow down disease progression towards ESKD.
